# Supplementary material for: Widespread reworking of Hadean-to-Eoarchean continents during Earth’s thermal peak
Source: Nat Commun. 2021 Jan 12;12:331. doi: 10.1038/s41467-020-20514-4 (PMC7803784; doi:10.1038/s41467-020-20514-4)
Supplement: Supplementary file 1 — Supplementary Information [file 41467_2020_20514_MOESM1_ESM.pdf]

Supplementary information for

Widespread reworking of Hadean-to-Eoarchean continents during Earth's thermal peak

Kirkland, C.L. [1], Hartnady, M.I.H. [1], Barham, M. [1], Olierook, H.K.H. [1], Steenfelt, A. [2], Hollis, J.A. [3]

[1]Timescales of Mineral Systems Group, School of Earth and Planetary Sciences, Curtin University, Perth, Western Australia 6102, Australia.

[2]The Geological Survey of Denmark and Greenland, Øster Voldgade 10, 1350 Copenhagen K, Denmark.

[3]Department of Geology, Ministry of Mineral Resources, Government of Greenland, P.O. Box 930, 3900 Nuuk, Greenland.

Corresponding author: Chris Kirkland (c.kirkland@curtin.edu.au)

Supplementary note 1

Regional geology: The study region in the northern North Atlantic Craton (NAC) is dominated by Mesoarchean tonalite-trondhjemite-granodioritie (TTG)-diorite gneisses with subsidiary intercalated mafic metavolcanic rocks, variably disrupted ultramafic rock complexes, and late-tectonic felsic to mafic igneous intrusions[1-4]. Eoarchean TTG gneisses occur in the southeast of the region and late Mesoarchean supracrustal rock sequences and Neoarchean granitoids occur in the north-western and south-eastern parts[5,6]. Magmatic rocks have dominant ages of c. 3230–3190 million years (Ma) and c. 3070–2970 Ma[6,7]. Near synchronous low-pressure granulite facies metamorphism and late-tectonic felsic magmatism followed these earlier magmatic episodes[3,8]. In a plate tectonic framework, the region has traditionally been interpreted to record accretion of a c. 3070 Ma magmatic arc to a c. 3200 Ma dioritic core, the generation of a significant volume of tonalitic crust, and subsequent reworking and intrusion of crustally-derived granodiorites and granites[9,10].

Næraa, et al.[11] interpreted zircon Hf compositions from TTG gneisses and (meta)sedimentary rocks from the North Atlantic Craton as indicating a transitional period 3500-3200 Ma ago, when the style of crustal evolution changed. The Hf evolution array they presented implied a common >3900 Ma old source reservoir, regarded as mafic. Post 3200 Ma zircon Hf signatures extended to both supra-chondritic as well as strongly sub-chondritic values, interpreted to imply plate tectonic processes by this stage in Earth history. In a study of zircon Hf compositions in TTG from the Mesoarchean part of the region, Gardiner, et al. [12] revealed a strikingly similar evolution pattern and noted the repeated involvement of an Eoarchean component in the growth of younger Mesoarchean crust. Furthermore, kimberlite dykes containing inherited zircon crystals up to 3.6 Ga also track an identical Hf evolution array consistent with an Eoarchean source either brought into place[13] or always present in the region. These Hf data sets, expressing a similar evolutionary pattern to that discussed in Næraa, et al.[11], led Gardiner, et al.[12] to highlight the potential of a volcanic plateau-type setting with repeated cycles of basaltic volcanism, burying existing crust to partial melting depths (i.e. 'vertical tectonics'). The protracted involvement of ancient crust was regarded as less easily reconciled in a subduction setting[14].

Supplementary note 2

U-Pb geochronology and Hf isotopic signature of stream sediments: Samples are presented from south to north through the Akia Terrane in the note below and in Supplementary table 1, which lists the number of analyses, the number within discordance limits, the detrital $^{207}Pb/^{206}Pb$ age peaks, the number of analyses contributing to such peaks, and the smallest fraction known (with 95% certainty) not to have been missed from the detrital population[15]. Supplementary table 1 also provides the number of Hf analyses within U-Pb discordance limits, a normality measure for the εHf_i distribution, the range in εHf_i values and model ages, and the kernel density model age peaks.

Sample 8: Four of 100 analyses in sample 8 are >10% discordant and not considered further. The remaining 96 analyses indicate significant $^{207}Pb/^{206}Pb$ age peaks at 3649 Ma, 3212 Ma, 3030 Ma, 2799 Ma, 2690 Ma, 2635 Ma and 2568 Ma, contributed to by 3, 7, 45, 10, 11, 9, and 8 analyses, respectively. For 96 dated grains, the fraction known with 95% certainty not to have been missed is 0.059. 31 Hf analyses on Sample 8 are within U-Pb discordance limits. εHfi values indicate the distribution of values is significantly different from normal (Shapiro-Wilk p = <0.05) with values ranging from –12.4 to 0.5 and a median of –2.3. Two stage model ages range from 4.03 to 3.15 Ga, with a kernel density fit indicating a dominant mode at c. 3.4 Ga.

Sample 9: Two of 100 analyses in sample 9 are > 10% discordant and not considered further. The remaining 98 analyses define significant age components at 3812 Ma, 3207 Ma, 3005 Ma, 2872 Ma and 2692 Ma, contributed to by 3, 24, 50, 3 and 3 analyses, respectively. For 98 dated grains, the fraction known with 95% certainty not to have been missed is 0.058. 48 Hf analyses on Sample 9 are within U-Pb discordance limits. Two analyses are mixtures (-93 and -100) and are not considered further. εHf_i values indicate the distribution of values is significantly different from normal (Shapiro-Wilk p = <0.05) with values ranging

from –13.5 to 4.6 and a median of –0.8. Two stage model ages range from 4.05 to 3.20 Ga, with a kernel density fit indicating a dominant mode at c. 3.4 Ga.

Sample 7: Two of 99 analyses in sample 7 are > 10% discordant and not considered further. The remaining 97 analyses define significant age components at 2992 Ma and 2850 Ma, contributed to by 81 and 4 analyses, respectively. For 97 dated grains, the fraction known with 95% certainty not to have been missed is 0.059. 26 Hf analyses on Sample 7 are within U-Pb discordance limits. εHf_i values indicate the distribution of values is significantly different from normal (Shapiro-Wilk p = <0.05) with values ranging from –10.5 to 1.6 and a median of –2.1. Two stage model ages range from 3.91 to 3.30 Ga, with a kernel density fit indicating a dominant mode at c. 3.4 Ga.

Sample 10: Thirteen of 100 analyses are > 10% discordant and not considered further. The remaining 87 analyses define a single significant age component at 3001 Ma, contributed to by 65 analyses. For 87 dated grains, the fraction known with 95% certainty not to have been missed is 0.064. 43 Hf analyses on Sample 10 are within U-Pb discordance limits and considered further. εHf_i values range from –15.2 to 2.6, with a median of –1.0. The Shapiro-Wilk test indicates the εHf_i values do not conform to a normal distribution. Two stage model ages range from 4.00 to 3.18 Ga, with a kernel density fit indicating a dominant mode at c. 3.3 Ga with a subsidiary mode at c. 3.9 Ga.

Sample 5: Three of 100 analyses are > 10% discordant and not considered further. The remaining 97 analyses define a single significant age component at 3029 Ma, contributed to by 85 analyses. For 97 dated grains, the fraction known with 95% certainty not to have been missed is 0.059. 25 Hf analyses on Sample 5 are within U-Pb discordance limits. εHf_i values indicate a parametric distribution (Shapiro-Wilk p = >0.1) with values ranging from –5.5 to 1.3 and a median of –1.2 (mean of –1.5). Two stage model ages range from 3.52 to 3.14 Ga, with a kernel density fit indicating a dominant mode at c. 3.35 Ga.

Sample 13: Three of 100 analyses are > 10% discordant and not considered further. The remaining 97 analyses define significant age components at 3014 Ma, 2792 Ma, 2703 Ma and 2567 Ma, contributed to by 70, 3, 5, 3 analyses, respectively. For 97 dated grains, the fraction known with 95% certainty not to have been missed is 0.059. 38 Hf analyses on Sample 13 are within U-Pb discordance limits. εHf_i values indicate a non-parametric distribution (Shapiro-Wilk p = <0.05) with values ranging from –19.9 to 0.8 and a median of –1.7. Two stage model ages range from 4.03 to 2.78 Ga, with a kernel density fit indicating a dominant mode at c. 3.3 Ga.

Sample 11: Three of 100 analyses are > 10% discordant and not considered further. The remaining 97 analyses define a single significant age component at 2988 Ma, contributed to by 76 analyses. For 97 dated grains, the fraction known with 95% certainty not to have been missed is 0.059. 39 Hf analyses on Sample 11 are within U/Pb discordance limits. εHf_i values indicate a non-parametric distribution (Shapiro-Wilk p = <0.05) with values ranging from –23.3 to 1.3 and a median of –0.8. Two stage model ages range from 3.44 to 2.82 Ga, with a kernel density fit indicating a dominant mode at c. 3.2 Ga.

Sample 3: All 100 analyses are within discordance limits. The analyses define significant age components at 2966 Ma and 2774 Ma, contributed to by 42 and 46 analyses, respectively. For 100 dated grains, the fraction known with 95% certainty not to have been missed is 0.057. 43 Hf analyses on Sample 3 are within U-Pb discordance limits. εHf_i values indicate the distribution of values is significantly different from normal (Shapiro-Wilk p = <0.05) with values ranging from –12.4 to 0.2 and a median of –7.9. Two stage model ages range from 3.72 to 2.41 Ga, with a kernel density fit indicating a dominant mode at c. 3.5 Ga.

Sample 12: Twelve of 97 analyses are > 10% discordant and not considered further. The remaining 85 analyses define significant age components at 3711 Ma, 3621 Ma, 3557 Ma, 2994 Ma, 2701 Ma and 2628 Ma contributed to by 8, 9, 7, 19, 14 and 7 analyses, respectively. For 85 dated grains, the fraction known with 95% certainty not to have been missed is 0.065. 59 Hf analyses on Sample 12 are within U-Pb discordance limits. One Hf analysis is imprecise and is excluded from further consideration. εHf_i values indicate a parametric distribution (Shapiro-Wilk p = >0.05) with values ranging from –22.0 to 3.62 and a

median of –6.8 (mean of -7.1). Two stage model ages range from 4.09 to 2.96 Ga, with a kernel density fit indicating a dominant mode at c. 3.9 Ga with a second peak at 3.4 Ga.

Sample 2: Fourteen of 101 analyses are > 10% discordant and not considered further. The remaining 87 analyses define significant age components at 3661 Ma, 2977 Ma and 2773 Ma contributed to by 3, 27 and 31 analyses, respectively. For 87 dated grains, the fraction known with 95% certainty not to have been missed is 0.064. 37 Hf analyses on Sample 2 are within U-Pb discordance limits. εHf_i values indicate a parametric distribution (Shapiro-Wilk p = >0.9) with values ranging from –18.8 to –0.3 and a median of –5.3 (mean of –6.8). Two stage model ages range from 3.97 to 3.29 Ga, with a kernel density fit indicating a dominant mode at c. 3.5 Ga.

Supplementary figure 1

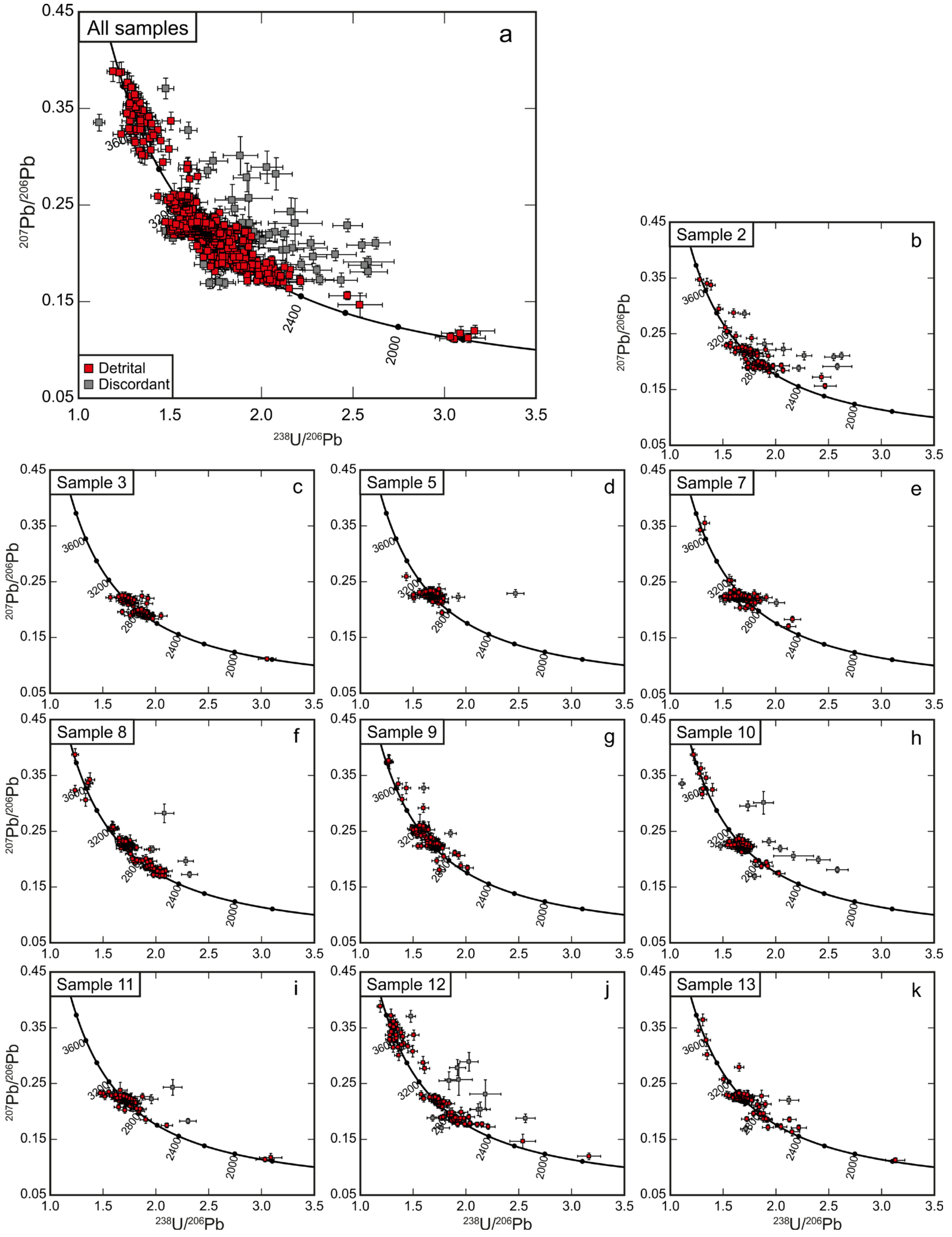

Supplementary Figure 1: Tera-Wasserburg concordia plots of U-Pb detrital zircon analyses from stream sediments in the North Atlantic Craton. a) All samples. b) Sample 2. c) Sample 3. d) Sample 5. e) Sample 7. f) Sample 8. g) Sample 9. h) Sample 10. i) Sample 11. j) Sample 12. k) Sample 13. Error bars are shown at the 95% confidence level.

Supplementary Table 1: Age and Hf population measures

Sample Id	N/(n)[a]	Age peaks[b]	95% certainty not missed[c]	εHf$_i$ n	εHf$_i$ range	Normality[d]	εHf$_i$ median	T_{DM}^2 range	T_{DM}^2 peak
8	100/96	3649 Ma, 3212 Ma, 3030 Ma, 2799 Ma, 2690 Ma, 2635 Ma and 2568 Ma, contributed to by 3, 7, 45, 10, 11, 9, and 8 analyses, respectively	0.059	31	-12.4 to 0.5	<0.05	-2.3	4.03 to 3.15 Ga	c. 3.4 Ga
9	100/98	3812 Ma, 3207 Ma, 3005 Ma, 2872 Ma, 2692 Ma, contributed to by 3, 24, 50, 3 and 3 analyses, respectively	0.058	48 (2 mixtures excluded)	-13.5 to 4.6	<0.05	-0.8	4.05 to 3.20 Ga	c. 3.4 Ga
7	99/97	2992 Ma and 2850 Ma, contributed to by 81 and 4 analyses, respectively	0.059	26	-10.5 to 1.6	<0.05	-2.1	3.91 to 3.30 Ga	c. 3.4 Ga
10	100/87	3001 Ma, contributed to by 65 analyses	0.064	43	-15.2 to 2.6	<0.05	-1.0	4.93 to 3.10 Ga	c. 3.3 Ga
5	100/97	3029 Ma, contributed to by 85 analyses	0.059	25	-5.5 to 1.3	>0.1	-1.2	3.52 to 3.14 Ga	c. 3.4 Ga
13	100/97	3014 Ma, 2792 Ma, 2703 Ma, 2567 Ma, contributed to by 70, 3, 5, 3 analyses, respectively	0.059	38	-19.9 to 0.8	<0.05	-1.7	4.03 to 2.78 Ga	c. 3.3 Ga
11	100/97	2988 Ma, contributed to by 76 analyses	0.059	39	-23.3 to 1.3	<0.05	-0.8	3.44 to 2.82 Ga	c. 3.2 Ga
3	100/100	2966 Ma and 2774 Ma, contributed to by 42 and 46 analyses, respectively	0.057	43	-12.2 to 0.2	<0.05	-7.9	3.72 to 2.41 Ga	c. 3.5 Ga
12	97/85	3711 Ma, 3621 Ma, 3557 Ma, 2994 Ma, 2701 Ma, and 2628 Ma contributed to by 8, 9, 7, 19, 14, and 7 analyses, respectively	0.065	59 (1 excluded)	-22.0 to 3.6	>0.05	-6.8	4.09 to 2.96 Ga	c. 3.4 Ga
2	101/87	3661 Ma, 2977 Ma, and 2773 Ma contributed to by 3, 27, and 31 analyses, respectively	0.064	37	-18.8 to -0.3	>0.9	-5.3	3.97 to 3.29 Ga	c. 3.5 Ga

[a]Number of analyses / number of analyses within 10% of concordia.[b]Age probability peaks and number of analyses that contribute to that peak. [c]95% not missed is the fraction not missed for the number of grains with accepted dates. [d]Normality is the Shapiro–Wilk test P value and is considered normal when P>0.05. [e]T_{DM}^2 is the two stage model age assuming a Lu/Hf ratio of 0.01 and the depleted model after Griffin et al., (2004).

Supplementary references

1 Garde, A. A. Accretion and evolution of an Archaean high-grade grey gneiss-amphibolite complex: the Fiskefjord area, southern West Greenland. (1997).

2 Garde, A. A. Thermal granulite-facies metamorphism with diffuse retrogression in Archaean orthogneisses, Fiskefjord, southern West Greenland. *Journal of Metamorphic Geology* **8**, 663-682 (1990).

3 Garde, A. A., Friend, C. R. L., Nutman, A. P. & Marker, M. Rapid maturation and stabilisation of middle Archaean continental crust: the Akia terrane, southern West Greenland. *Bulletin of the Geological Society of Denmark* **47**, 1-27 (2000).

4 Szilas, K., Kelemen, P. B. & Bernstein, S. Peridotite enclaves hosted by Mesoarchaean TTG-suite orthogneisses in the Fiskefjord region of southern West Greenland. *GeoResJ* **7**, 22-34 (2015).

5 Kirkland, C. L., Yakymchuk, C., Hollis, J., Heide-Jørgensen, H. & Danišík, M. Mesoarchean exhumation of the Akia terrane and a common Neoarchean tectonothermal history for West Greenland. *Precambrian Research* **314**, 129-144, doi:https://doi.org/10.1016/j.precamres.2018.06.004 (2018).

6 Friend, C. R. L. & Nutman, A. P. Tectono-stratigraphic terranes in Archaean gneiss complexes as evidence for plate tectonics: The Nuuk region, southern West Greenland. *Gondwana Research* **72**, 213-237 (2019).

7 Garde, A. A., Dyck, B., Esbensen, K. H., Johansson, L. & Möller, C. The Finnefjeld domain, Maniitsoq structure, West Greenland: Differential rheological features and mechanical homogenisation in response to impacting? *Precambrian Research* **255**, 791-808 (2014).

8 Yakymchuk, C. *et al.* Mesoarchean partial melting of mafic crust and tonalite production during high-T–low-P stagnant tectonism, Akia Terrane, West Greenland. *Precambrian Research*, 105615, doi:https://doi.org/10.1016/j.precamres.2020.105615 (2020).

9 Garde, A. A. A mid-Archaean island arc complex in the eastern Akia terrane, Godthåbsfjord, southern West Greenland. *Journal of the Geological Society* **164**, 565-579, doi:10.1144/0016-76492005-107 (2007).

10 Garde, A. A., McDonald, I., Dyck, B. & Keulen, N. Searching for giant, ancient impact structures on Earth: The Mesoarchaean Maniitsoq structure, West Greenland. *Earth and Planetary Science Letters* **337-338**, 197-210, doi:https://doi.org/10.1016/j.epsl.2012.04.026 (2012).

11 Næraa, T. *et al.* Hafnium isotope evidence for a transition in the dynamics of continental growth 3.2 Gyr ago. *Nature* **485**, 627-630 (2012).

12 Gardiner, N. J. *et al.* Building Mesoarchaean crust upon Eoarchaean roots: the Akia Terrane, West Greenland. *Contributions to Mineralogy and Petrology* **174**, 20 (2019).

13 Gardiner, N. J. *et al.* North Atlantic Craton architecture revealed by kimberlite-hosted crustal zircons. *Earth and Planetary Science Letters* **534**, 116091 (2020).

14 Johnson, T. E., Brown, M., Gardiner, N. J., Kirkland, C. L. & Smithies, R. H. Earth's first stable continents did not form by subduction. *Nature* **543**, 239, doi:10.1038/nature21383 (2017).

15 Vermeesch, P. How many grains are needed for a provenance study. *Earth and Planetary Science Letters* **224**, 441-451 (2004).
