# Supplementary material for: Widespread reworking of Hadean-to-Eoarchean continents during Earth’s thermal peak
Source: Nat Commun. 2021 Jan 12;12:331. doi: 10.1038/s41467-020-20514-4 (PMC7803784; doi:10.1038/s41467-020-20514-4)
Supplement: Supplementary file 3 — Description of Additional Supplementary Files [file 41467_2020_20514_MOESM3_ESM.pdf]

Captions for supplementary data files.

Supplementary data 1:

Zircon U-Pb ratios and ages for stream sediment samples of the North Atlantic Region.

Supplementary data 2:

Zircon Lu-Hf isotopic data for stream sediment samples of the North Atlantic Region.

Supplementary data 3:

Compilation of U-Pb and Lu-Hf isotopic ratios on reference materials during analytical sessions of this study.

Supplementary data 4:

Compilation of zircon cathodoluminescence images.

Supplementary data 5:

Source data file of zircon Hf isotope statistical parameters for different continents.
