# Supplementary figures and images for: Widespread reworking of Hadean-to-Eoarchean continents during Earth’s thermal peak
Source: Nat Commun. 2021 Jan 12;12:331. doi: 10.1038/s41467-020-20514-4 (PMC7803784; doi:10.1038/s41467-020-20514-4)

## Sample 2

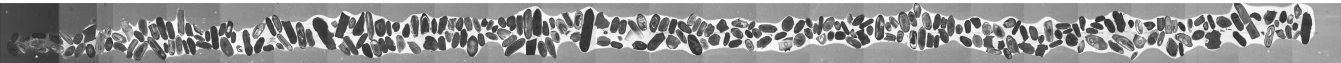

# Sample 3

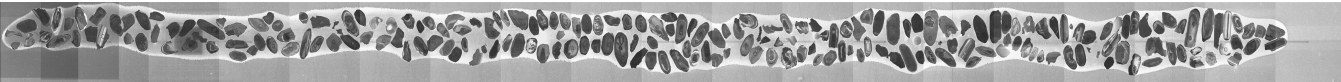

# Sample 5

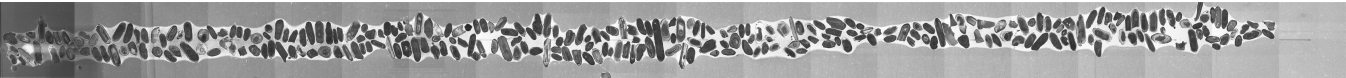

# Sample 7

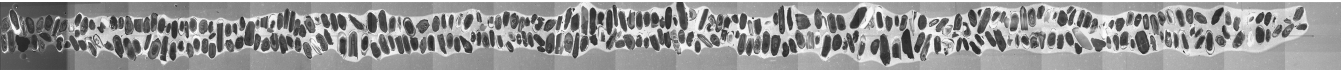

# Sample 8

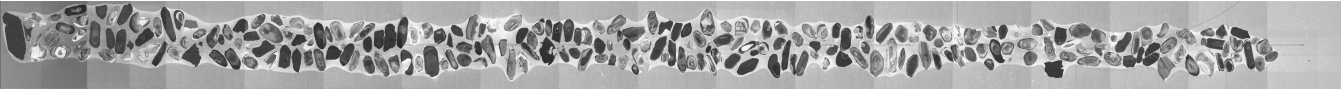

Sample 9

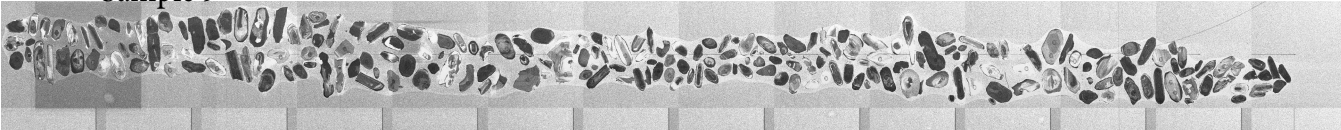

Sample 10

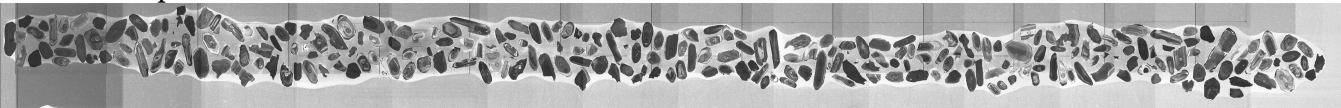

Sample 11

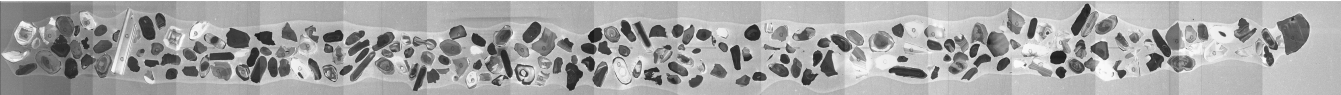

Sample 12

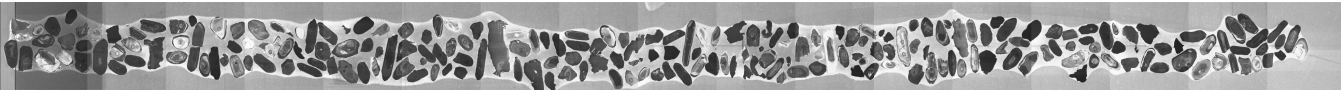

Sample 13

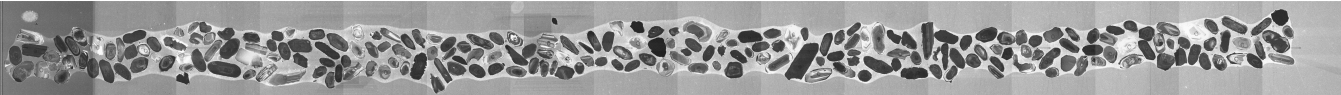

Supplement: Supplementary file 7 — Supplementary Data 4 [file 41467_2020_20514_MOESM7_ESM.zip › Supplementary Data 4.pdf]
